# Downstream effects of plectin mutations in epidermolysis bullosa simplex with muscular dystrophy

**DOI:** 10.1186/s40478-016-0314-7

**Published:** 2016-04-27

**Authors:** Lilli Winter, Matthias Türk, Patrick N. Harter, Michel Mittelbronn, Cornelia Kornblum, Fiona Norwood, Heinz Jungbluth, Christian T. Thiel, Ursula Schlötzer-Schrehardt, Rolf Schröder

**Affiliations:** Institute of Neuropathology, Friedrich-Alexander University Erlangen-Nürnberg (FAU), Schwabachanlage 6, 91054 Erlangen, Germany; Department of Neurology, Friedrich-Alexander University Erlangen-Nürnberg (FAU), Erlangen, Germany; Institute of Neurology (Edinger Institute), Goethe University Frankfurt, Frankfurt, Germany; Department of Neurology, University Hospital of Bonn, Bonn, Germany; Center for Rare Diseases Bonn (ZSEB), University Hospital of Bonn, Bonn, Germany; Department of Neurology, Ruskin Wing, King’s College Hospital, London, UK; Department of Paediatric Neurology, Neuromuscular Service, Evelina Children’s Hospital, St Thomas’ Hospital, London, UK; Randall Division of Cell and Molecular Biophysics, Muscle Signalling Section, King’s College, London, UK; Department of Basic and Clinical Neuroscience, IoPPN, King’s College, London, UK; Institute of Human Genetics, Friedrich-Alexander University Erlangen-Nürnberg (FAU), Erlangen, Germany; Department of Opthalmology, Friedrich-Alexander University Erlangen-Nürnberg (FAU), Erlangen, Germany

**Keywords:** Plectin, Epidermolysis bullosa simplex with muscular dystrophy, Skeletal muscle, Intermediate filaments, Mitochondria, Desmin, Protein aggregates

## Abstract

Mutations of the human plectin gene *(PLEC)* on chromosome 8q24 cause autosomal recessive epidermolysis bullosa simplex with muscular dystrophy (EBS-MD). In the present study we analyzed the downstream effects of *PLEC* mutations on plectin protein expression and localization, the structure of the extrasarcomeric desmin cytoskeleton, protein aggregate formation and mitochondrial distribution in skeletal muscle tissue from three EBS-MD patients.

*PLEC* gene analysis in a not previously reported 35-year-old EBS-MD patient with additional disease features of cardiomyopathy and malignant arrhythmias revealed novel compound heterozygous (p.(Phe755del) and p.(Lys1040Argfs*139)) mutations resulting in complete abolition of plectin protein expression. In contrast, the other two patients with different homozygous *PLEC* mutations showed preserved plectin protein expression with one only expressing rodless plectin variants, and the other markedly reduced protein levels. Analysis of skeletal muscle tissue from all three patients revealed severe disruption of the extrasarcomeric intermediate filament cytoskeleton, protein aggregates positive for desmin, syncoilin, and synemin, degenerative myofibrillar changes, and mitochondrial abnormalities comprising respiratory chain dysfunction and an altered organelle distribution and amount.

Our study demonstrates that EBS-MD causing *PLEC* mutations universally result in a desmin protein aggregate myopathy phenotype despite marked differences in individual plectin protein expression patterns. Since plectin is the key cytolinker protein that regulates the structural and functional organization of desmin filaments, the defective anchorage and spacing of assembled desmin filaments is the key pathogenetic event that triggers the formation of desmin protein aggregates as well as secondary mitochondrial pathology.

**Electronic supplementary material:**

The online version of this article (doi:10.1186/s40478-016-0314-7) contains supplementary material, which is available to authorized users.

## Introduction

Plectin, a multi-domain protein of exceptionally large size (>500 kDa), is abundantly expressed in a wide range of mammalian cells and tissues, most prominently in muscle, brain and stratified squamous epithelia [20]. It acts as a multi-functional linker protein and signalling scaffold that centrally orchestrates the structural and functional organization of filamentous cytoskeletal networks, thereby contributing to fundamental biomechanical properties of mechanical stress-bearing tissue [21]. The essential role of plectin in the latter is highlighted by the observation that mutations in the human plectin gene (*PLEC*) on chromosome 8q24 cause a variety of rare human disorders (referred to as “plectinopathies”), namely autosomal recessive epidermolysis bullosa simplex with muscular dystrophy (EBS-MD, MIM #226670), EBS-MD with myasthenic features (EBS-MD-MyS), limb girdle muscular dystrophy type 2Q (LGMD2Q, MIM #613723), EBS with pyloric atresia (EBS-PA, MIM #612138), and the autosomal dominant variant EBS-Ogna [23, 25]. While the skin blistering in plectinopathies has been attributed on the molecular level to a disruption of the normal keratin intermediate filament (IF) anchorage to hemidesmosomes [21], the downstream effects of plectin mutations in striated muscle are less clearly defined and to date have only been addressed in very few detailed studies [2, 17].

Here we report the clinical, genetic, myopathological, and biochemical findings in a not previously reported German EBS-MD patient with additional features of cardiomyopathy and malignant arrhythmias. Furthermore, we analyse the downstream effects of his novel disease-causing compound heterozygous *PLEC* mutations and two other homozygous *PLEC* mutations on i) plectin protein expression, ii) the structure of the extrasarcomeric desmin cytoskeleton, iii) protein aggregate formation and iv) the subcellular distribution and biochemical properties of mitochondria in skeletal muscle tissue.

## Materials and methods

### Patients

**Fig. 1:**
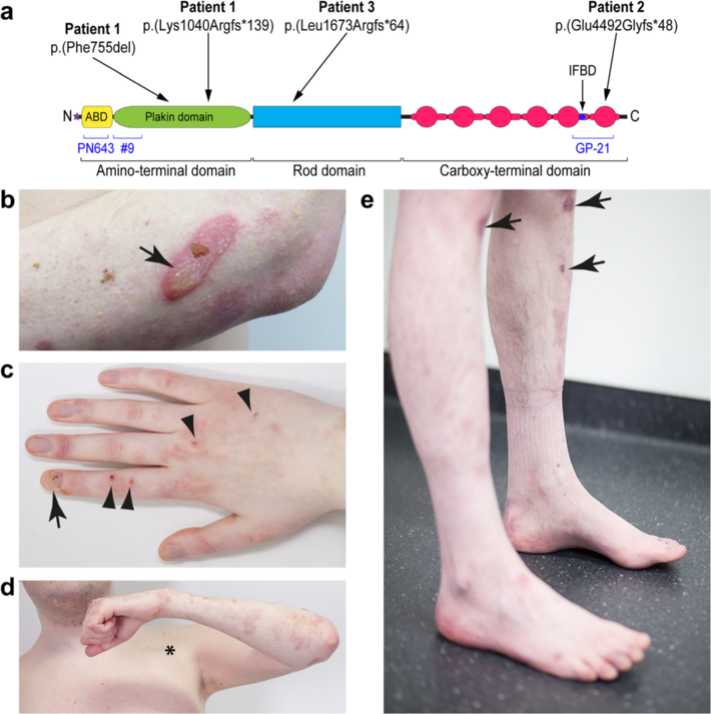
Schematic representation of the localization of the *PLEC* mutations identified and clinical features of EBS-MD. **a** Schematic domain map of plectin and positional mapping of the EBS-MD mutations studied in this work. The tripartite structure of the plectin molecule comprises a central, α-helical rod domain (*blue*), which is flanked by N- and C-terminal globular domains. The N-terminal domain harbors an actin-binding domain (ABD, *yellow*) and a plakin domain (*green*), whereas the C-terminal domain consists of six highly homologous plectin repeat domains (*red*), harboring an intermediate filament-binding domain (IFBD) between repeat 5 and 6. Note that EBS-MD 1 is compound heterozygous, whereas EBS-MD2 and 3 are homozygous mutations. Binding sites of antibodies PN643 and #9, recognizing plectin’s N-terminal region, and GP-21 antibodies, recognizing plectin’s C-terminal region, are indicated in blue. **b** Large erythematous skin blister (*arrow*) on the proximal forearm of patient 1. **c** Nail dystrophy of the right index finger (*arrow*) and small skin blisters (*arrowheads*) of patient 1. **d** Muscle atrophy of the upper extremity in patient 1. Asterisk indicates the scar from the implantation of the cardioverter defibrillator. **e** Distal muscle atrophy of the lower extremities with inability to stand on heels. Note the multiple skin lesions of patient 1 (*arrows*)

This study was approved by the local Ethics committees of each participating institution. Written informed consent was obtained from all participants. Patient 1 is a not previously reported, 35-year-old male patient of German origin with EBS-MD. Patient 2 is a previously reported German female with EBS-MD due to a homozygous 16-bp insertion frameshift-mutation c.13459_13474dup (NM_000445.3) in the 3′end of exon 32 of the *PLEC* gene, causing a premature termination codon (p.(Glu4492Glyfs*48)) [17] (see also Fig. 1a). She had last been seen for clinical evaluation at the age of 25 years but subsequently had tragically died in a domestic fire accident. Patient 3 is a 24-year-old British female with EBS-MD caused by a homozygous 19-bp deletion frameshift-mutation c.5018_5036del (NM_000445.3) in exon 31, which causes a premature termination codon (p.(Leu1673Argfs*64)) [11] (see also Fig. 1a). See Table 1 for more detailed clinical information of all three patients.

**Table 1:** Genetic, clinical and myopathological features of EBS-MD patients

Pat.	*PLEC*-Mutations and their effects on plectin protein expession	Clinical phenotype	Myopathological findings
1, m	▪ compound heterozygous ▪ exon 19: c.2264_2266delTCT/p.(Phe755del) (in-frame deletion) ▪ exon 24: c.3119_3120delAA/p.(Lys1040Argfs*139) (premature termination codon) ➢ no detectable plectin protein expression	▪ EBS: since birth ▪ myopathy: generalized; predominantly affecting shoulder-girdle and lower leg muscles ▪ dilated cardiomyopathy; ventricular arrhythmias	▪ LM: myopathic pattern; fibers with rubbed-out lesions; desmin-protein aggregates ▪ EM: degenerating myofibrills; autophagic vacuoles (triceps muscle; age at biopsy: 33 years)
2, f [17]	▪ homozygous ▪ exon 32: c.13459_13474dup/p.(Glu4492Glyfs*48) (premature termination codon) ➢ reduced plectin protein expression (full-length and rodless)	▪ EBS: since birth ▪ myopathy: generalized; predominantly affecting proximal muscle groups; facial weakness; marked bilateral ptosis; incomplete external ophthalmoplegia, ▪ mild left ventricular cardiac hypertrophy ▪ bilateral cataracts ▪ brain atrophy with hydrocephalus ex vacuo	▪ LM: myopathic pattern; rimmed-vacuoles; fibers with rubbed-out lesions; desmin-protein aggregates ▪ EM: degenerating myofibrils; cytoplasmic bodies; abnormally shaped mitochondria with paracristalline inclusions; desmin-positive filaments and sub-sarcolemmal protein aggregates (quadriceps muscle; age at biopsy: 25 years)
3, f [11]	▪ homozygous ▪ exon 31: c.5018_5036del/p.(Leu1673Argfs*64) (premature termination codon) ➢ expression of rodless plectin protein only	▪ EBS: since birth ▪ myopathy: mild neck flexion, shoulder abduction, elbow flexion and hand muscle weakness ▪ inspiratory stridor in the postnatal period due to moderate supraglottic obstruction caused by interarytenoid scarring	▪ LM: myopathic pattern; fibers with rubbed-out lesions; COX-negative fibers; desmin-protein aggregates ▪ EM: not performed (biceps muscle; age at biopsy 24 years)

Plectin mutations are assigned to a common reference sequence, GenBank accession number NM_000445.3. Pat. indicates patient; m, male; f, female; yrs, years; LM, light microscopy; EM, electron microscopy

### Genetic studies

DNA of patient 1 and his parents was fragmented and enriched using the TruSight one enrichment kit (Illumina, San Diego, USA). Sequencing was performed on a MiSeq (Illumina, San Diego, USA) in paired-end reads (2× 200 bp). After duplicate removal and quality control the sequence reads were mapped with the BWA aligner [9] to the reference genome (hg19). Variants were called using 5 different calling programs (GATK, SNVer, CASAVA, SOAPSNP and SOAPindel) and filtered based on population frequency (<0.001 in 94 in house controls and exomes of the ExAC consortium), position (exon and splice site) and mutational effect (CADD score, Polyphen2, SIFT, MutationTaster). Graphical presentation of the mapped sequences was viewed with the Integrative Genomics Viewer (IGV) [14]. The resulting variants were Sanger sequenced and the segregation in the family confirmed.

### Muscle biopsies and myopathological analyses

Patient 1 had a diagnostic muscle biopsy from his left triceps brachii muscle in 2013. Skeletal muscle tissue (left quadriceps) from patient 2 obtained as part of the diagnostic process in 2001 was reevaluated. Patient 3 underwent a muscle biopsy from her left biceps humeri in 2014. Skeletal muscle tissue was used for histochemical studies by standard methods [4]. Immunofluorescence microscopy was performed on frozen muscle sections (5 μm) using guinea pig antiserum (AS) to plectin (Progen, GP-21; recognizing the C-terminal domain of plectin, aa 4367–4684 in exon 32), mouse monoclonal antibodies (mAbs) to plectin (KeraFAST, PN643; recognizing the N-terminal actin binding domain, aa 171–595), mAbs to desmin (Dako, clone D33), rabbit AS to synemin 3 (D. Paulin, France, [26]), goat AS to syncoilin (Santa Cruz Biotechnology Inc., sc-162284), and mAbs to complex IV (subunit I, Invitrogen, #459600) in combination with donkey anti-guineapig IgG Alexa Fluor 555, goat anti-mouse IgG Alexa Fluor 488, donkey anti-mouse IgG Alexa Fluor 555, donkey anti-rabbit IgG Alexa Fluor 488, and donkey anti-goat IgG Alexa Fluor 488 (all from Life Technologies). Images were acquired using a Zeiss LSM 780 confocal laser scanning microscope.

### Western blot analyses

Skeletal muscle tissue was homogenized in lysis buffer (pH 6.8) containing 5 mM Tris, 10 % SDS, 0.2 M DTT, 1 mM EDTA, 100 mM NaF, 50 mM ß-glycerphosphate, 2 mM Na_3_VO_4_, 1 mM PMSF, and Complete mini protease inhibitor cocktail (Roche) using a TissueLyser II (Qiagen; 30×/s, 2 min; three repetitions). The homogenate was centrifuged for 10 min at 13,000 rpm and the supernatant was stored at −20 °C. For gel electrophoresis, the tissue homogenate was mixed with SDS-sample buffer, denatured at 95 °C for 5 min, and loaded onto 4–15 % Mini-PROTEAN® TGX™ Precast Gels (BioRad). Proteins were transferred onto nitrocellulose membranes, and the following primary antibodies were used for immunoblot analyses: guinea pig AS to plectin (Progen, GP-21; recognizing the C-terminal domain of plectin, aa 4367–4684 in exon 32), rabbit AS to plectin (#9, G. Wiche, Austria; recognizing plectin exons 9–12, [1]), rabbit AS to desmin (Abcam, ab8592), rabbit AS to synemin 3 (D. Paulin, France, [26]), goat AS to syncoilin (Santa Cruz Biotechnology Inc., sc-162284), mAbs to α-actinin (Sigma, #7811), mAbs to GAPDH (Sigma, G8795), mAbs to complex II (70 kDa Fp Subunit, Invitrogen, # 459200), mAbs to complex V (α-subunit, Invitrogen, #439800). For detection, HRP-conjugated secondary antibodies (all from BioRad) in combination with Pierce SuperSignal West Pico Chemiluminescent Substrate (Thermo Scientific) were used.

## Results

### Novel compound heterozygous *PLEC* mutations lead to EBS-MD plus cardiomyopathy and life-threatening arrhythmias

Patient 1 is a 35-year-old German male with a past medical history of skin blistering (Fig. 1b) and nail dystrophy (Fig. 1c) since birth. At the age of 27 he first experienced recurrent episodes of dizziness and sudden loss of consciousness. Cardiological evaluation at that time revealed a dilated cardiomyopathy with markedly reduced left ventricular ejection fraction (20–30 %), as well as episodes characterized by self-limiting ventricular fibrillation and torsades. Since the cardiac arrhythmias were graded as life-threatening, a single-chamber cardioverter defibrillator was implanted (see asterisk in Fig. 1d). Slowly progressive muscle weakness and myalgia were first noted soon after the manifestation of cardiac problems. Neurological examination at the age of 32 revealed muscle weakness and wasting predominantly affecting shoulder-girdle and lower leg muscles with an associated marked steppage gait (Fig. 1d and e). Needle electromyography revealed a myopathic pattern in proximal and distal muscles of both the upper and lower limbs. CK levels were markedly elevated (up to 3782 IU/l). *PLEC* sequencing revealed two novel heterozygous variants, a maternally inherited in-frame deletion c.2264_2266delTCT/p.(Phe755del) in exon 19 and a paternally inherited frameshift deletion c.3119_3120delAA/p.(Lys1040Argfs*139) in exon 24 (see Additional file 1: Figure S1 and Table 1). Both are classified as pathogenic according to the ACMG criteria and are therefore considered disease causing variants.

### Impact of *PLEC* mutations on skeletal muscle morphology and plectin protein levels: no plectin, less plectin, or expression of rodless plectin

**Fig. 2:**
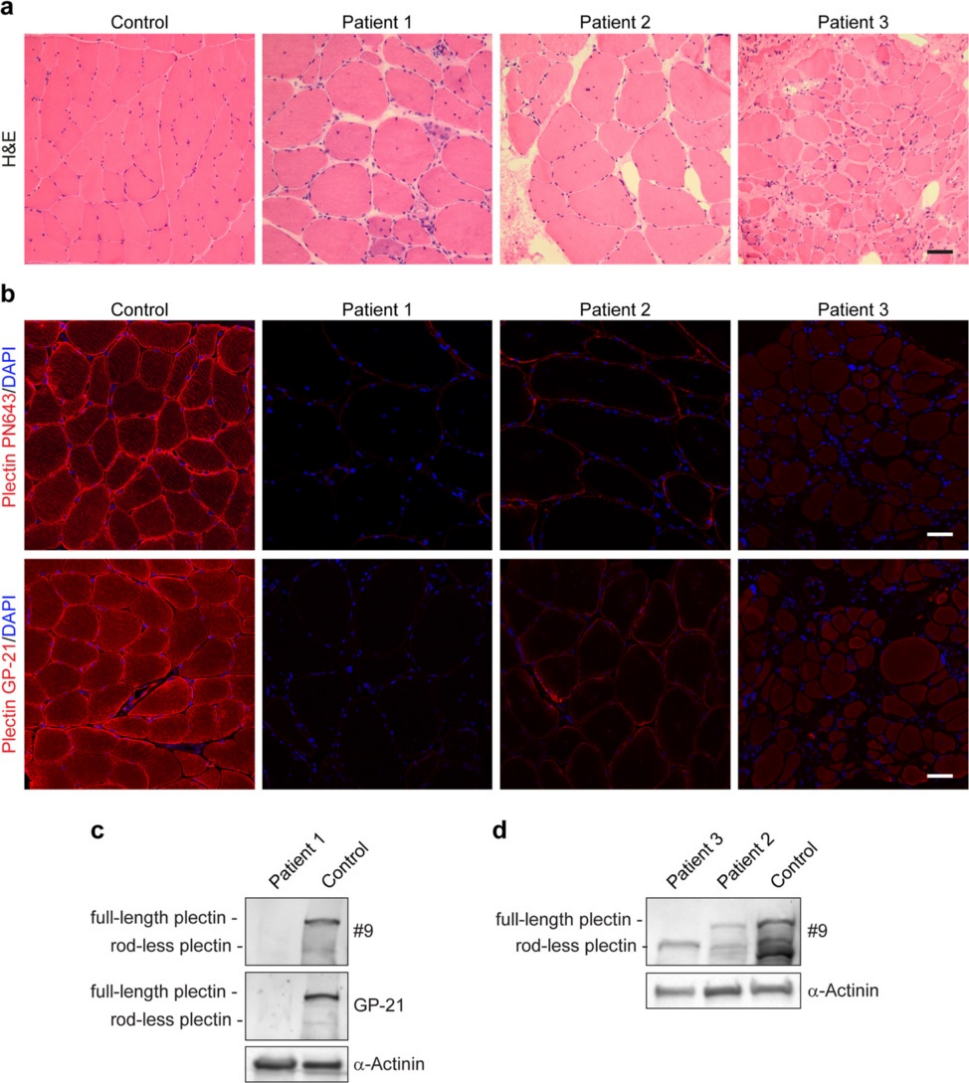
Skeletal muscle pathology and plectin expression in EBS-MD muscle. **a** Skeletal muscle sections from a healthy control and patients 1–3 were stained by hematoxylin and eosin. Note the rounding of muscle fibers with marked variation in fiber size, internalization and clustering of myonuclei, and increased amount of connective tissue in EBS-MD patient muscles. Scale bar: 50 μm. **b** Immunofluorescence microscopy of skeletal muscle sections from a healthy control and patients 1–3 using antibodies PN643, regognizing the N-terminal actin binding domain (aa 171–595), and GP-21, recognizing plectin’s C-terminus (aa 4367–4684 in exon 32). Nuclei were visualized using Dapi. Note the drastically reduced plectin staining intensity in muscle tissue from patients 2 and 3, as well as the completely absent staining in patient 1. Scale bar: 50 μm. **c** and **d** Immunoblotting of cell lysates prepared from patient 1 (**c**), patients 2 and 3 (**d**), and a healthy control. Antibodies used for detection are indicated. GAPDH was used as loading control. Note that plectin antibody GP-21 detects plectin’s C-terminal region (aa 4367–4684 in exon 32), whereas plectin antiserum #9 recognizes the N-terminal region (exons 9–12). While no plectin band could be detected in patient 1, markedly reduced but still recognizable plectin levels were observed in patient 2. Patient 3 had no expression of full-length plectin, while rodless plectin was still found

Myopathological analyses of skeletal muscle specimens from all three EBS-MD patients with genetically confirmed *PLEC* mutations revealed a marked myodegenerative pattern with increased endomysial connective and fatty tissue, highly variable fiber size diameters (5 to 145 μm), predominance of type 1 fibers, fiber splitting as well as de- and regenerating fibers (Fig. 2a). Quantification of muscle fibers with internally located myonuclei demonstrated an abnormal myonuclear positioning in 47 % of fibers in patient 1, in 55 % of fibers in patient 2 and in 10 % of fibers in patient 3. Muscle biopsy from patient 2 additionally showed multiple muscle fibers with rimmed vacuoles (data not shown). To investigate the effects of the three different *PLEC* mutations on plectin protein expression in skeletal muscle, we performed indirect immunofluorescence microscopy and immunoblot analyses using plectin-specific antibodies (Fig. 2b-d). These analyses showed a marked variation of plectin protein levels among the three EBS-MD cases. Immunofluorescence analysis using antibodies directed against the N-terminal or the C-terminal part of plectin demonstrated the complete absence of specific immunostaining in patient 1, whereas the other two EBS-MD muscle specimens displayed markedly reduced plectin staining in comparison to normal controls (Fig. 2b). Plectin immunoblot analysis of normal human skeletal muscle detected two bands, one with a strong signal intensity corresponding to a molecular mass of approximately 500 kDa and a second, very faint signal at 390 kDa. While the former represents the full length plectin protein species, the latter corresponds to rodless plectin variants [13]. Analysis of patient 1 showed a complete absence of plectin, whereas patient 2 displayed a marked reduction in the signal intensities of both bands. In contrast, analysis of the muscle probe from patient 3 resulted in the sole detection of rodless plectin variants (Fig. 2c and d, and Table 1). Thus, the *PLEC* mutations in our three EBS-MD patients led to marked individual differences in plectin protein expression in skeletal muscle.

### Aberrant plectin expression leads to destruction of the desmin network and desmin protein aggregation pathology

**Fig. 3:**
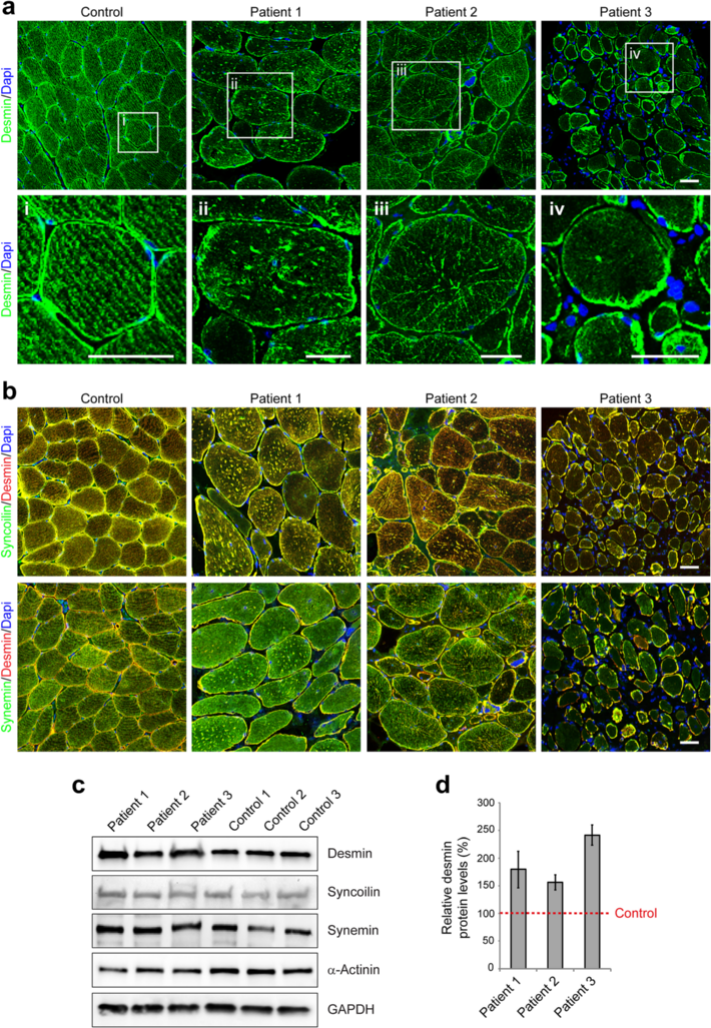
Disruption and aggregation of IF networks in EBS-MD muscle. **a** Confocal imaging of desmin-stained skeletal muscle specimens from a healthy control and EBS-MD patients. Panels i-iv are magnifications of the boxed areas in panel a. Note the formation of desmin-positive protein aggregates in all EBS-MD samples. Scale bars: 50 μm (**a**), 25 μm (panels i-iv). **b** Skeletal muscle sections were co-stained for desmin and synemin or syncoilin. Note that all three types of IFs lose their proper orientation in EBS-MD muscles and co-accumulate in desmin-positive protein aggregates. Scale bar: 50 μm. **c** Immunoblotting of cell lysates prepared from EBS-MP patients and three healthy controls. Antibodies used for detection are indicated. GAPDH and α-actinin were used as loading controls. **d** Signal intensities of desmin, syncoilin and synemin protein bands as shown in (**c**) were densitometrically measured and normalized to the total protein content (assessed by GAPDH staining). Healthy controls (*dashed line*) are set at 100 %. Mean values ± SEM, 3 replicates

One major function of plectin in skeletal muscle is the proper organization and anchorage of the three-dimensional, extrasarcomeric IF network, which – in addition to plectin - is mainly composed of the IF proteins desmin, syncoilin, synemin [7, 16]. In contrast to the regular IF staining pattern in normal controls, our immunofluorescence analyses on muscle tissue from EBS-MD patients using corresponding antibodies demonstrated a marked disruption of the endogenous desmin network in conjunction with subsarcolemmal and sarcoplasmic desmin-, syncoilin-, and synemin-positive protein aggregates in nearly all muscle fibers (Fig. 3a and b). Subsequent immunoblot analyses of all three EBS-MD probes revealed significantly increased desmin protein levels (180 % in patient 1; 156 % in patient 2 and 242 % in patient 3), whereas the levels of syncoilin and synemin were unchanged (Fig. 3c and d). Thus, irrespective of the individual *PLEC* mutations and their corresponding plectin protein expression pattern, these patients universally exhibited destruction of the desmin filament network and desmin-positive protein aggregates, which are the hallmarks of the EBS-MD muscle pathology.

### From cytoskeletal to mitochondrial pathology in EBS-MD muscle tissue

**Fig. 4:**
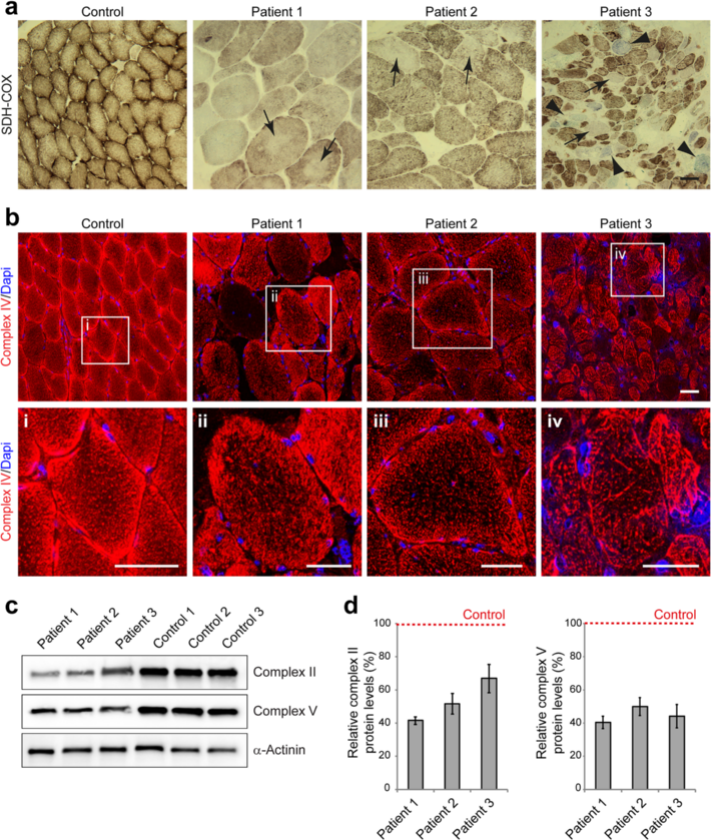
Mitochondrial alterations in EBS-MD muscle. **a** Skeletal muscle specimens from a healthy control and EBS-MD patients were histologically double-stained for SDH and COX. Note the presence of “rubbed-out” areas (*arrows*), and the presence of COX-negative fibers in patient 3 (*arrowheads*). Scale bar: 50 μm. **b** Confocal imaging of mitochondrial respiratory complex IV-stained skeletal muscle specimens from a healthy control and EBS-MD patients. Panels i-iv are magnifications of the boxed areas in panel b. Note the reduced staining intensity in all EBS-MD samples. Scale bars: 50 μm (**b**), 25 μm (panels i-iv). **c** Immunoblotting of cell lysates prepared from EBS-MD patients and three healthy controls using antibodies for mitochondrial respiratory complex II or V. α-Actinin was used as loading control. **d** Signal intensities of respiratory complex II or V protein bands as shown in (**c**) were densitometrically measured and normalized to the total protein content (assessed by α-actinin staining). Healthy controls (*dashed line*) are set to 100 %. Mean values ± SEM, 3 replicates

Structural and functional alterations of mitochondrial networks have previously been described in skeletal muscle specimens from EBS-MD patients [10, 17] as well as muscle-restricted conditional plectin knockout mice [7]. Succinate dehydrogenase (SDH) and cytochrome oxidase (COX) stains of muscle specimens from our EBS-MD patients clearly demonstrated an abnormal pattern with less ordered, often coarse, and thread-like appearing mitochondrial networks (Fig. 4a). Moreover, in a subset of fibers, large sarcoplasmic areas with markedly attenuated enzyme staining (“rubbed-out” lesions) became apparent (Fig. 4a, arrows). In addition, several COX-negative/SDH-positive fibers were identified in the muscle biopsy from patient 3 (Fig. 4a, arrowheads). However, no classical ragged-red fibers were seen in Gomori-Trichrome stains (data not shown). In keeping with the enzymatic COX stains, immunofluorescence analysis using a complex IV antibody showed reduced signal intensities in all EBS-MD samples (Fig. 4b). Furthermore, immunoblotting with antibodies directed against complex II and V of respiratory chain enzymes revealed a dramatic decrease of the respective expression levels in all EBS-MD samples (Fig. 4c). Expression levels of complex II were reduced to 42 % (patient 1), 52 % (patient 2), or 67 % (patient 3), and complex V to to 41 % (patient 1), 50 % (patient 2), or 44 % (patient 3) compared to control levels (Fig. 4d). In addition to changes in the subcellular distribution of mitochondria, the decrease in the levels of complex II, IV and V suggest a reduction in the total amount of mitochondria in EBS-MD muscle tissue.

## Discussion

Our genetic analysis in patient 1 identified two novel compound heterozygous *PLEC* mutations. In addition to the skin and skeletal muscle involvement characteristic of the disease, this patient developed a dilated cardiomyopathy and life-threatening episodes of cardiac arrhythmias necessitating the implantation of a single-chamber cardioverter defibrillator. Plectin-related cardiac disease comprising mild left ventricular hypertrophy [17], reduced ejection fraction in combination with artrial fibrillation [3], dilated cardiomyopathy [2], and left ventricular non-compaction cardiomyopathy [19] have previously been described in a subset of EBS-MD patients. However, this is the first report indicating that even life-threatening cardiac disease manifestations may occur before the onset of skeletal muscle symptoms, suggesting that regular cardiological assessments including electrophysiological and cardiac imaging studies should be part of the diagnostic work-up of all EBS-MD patients.

To characterize the downstream effects of *PLEC* mutations, we analysed skeletal muscle tissue from the newly-reported patient in comparison to muscle biopsies from two other previously reported EBS-MD patients, in whom the homozygous disease-causing mutations reside in exon 31 and exon 32 of the *PLEC* gene, respectively. Our immunoblot studies demonstrated a heterogenous pattern of plectin expression with complete abolition of plectin protein expression in patient 1, markedly reduced plectin levels in patient 2, and the sole presence of rodless plectin variants in patient 3. Rodless plectin isoforms, lacking the central rod domain due to alternative splicing of exon 31 [5], are expressed in normal human skeletal muscle and skin tissue, though in relatively low amounts [8, 16]. A recent study in plectin knock-in mice indicated that rodless plectin might functionally compensate for the loss of full-length plectin in murine skin and skeletal muscle tissue [6]. However, this notion is clearly contradicted by our findings in patient 3, who - despite expressing rather high levels of rodless plectin in muscle – developed a severe form of muscular dystrophy.

Irrespective of the individual *PLEC* mutations and their varying consequences on plectin protein expression, our analyses revealed a rather uniform picture of EBS-MD muscle pathology, characterized by degenerative myofibrillar changes, subsarcolemmal and sarcoplasmic desmin-positive protein aggregates, and mitochondrial abnormalities. Since these myopathological alterations are also the morphological hallmark of myofibrillar myopathies [15, 18], EBS-MD can thus be classified as a subtype of these protein aggregate myopathies.

Studies in primary human differentiating skeletal muscle cells and various transgenic plectin mouse models indicate that plectin exerts a pivotal role in the structural and functional organization of the three-dimensional desmin IF network, with an essential role in mechanosensing and in protecting skeletal muscle cells against mechanical stress [7, 16, 24]. Our study demonstrates universal disruption of the endogenous desmin-, synemin-, and syncoilin-IF networks in nearly all EBS-MD skeletal muscle fibers. Hence, the faulty subcellular spacing and anchorage of preformed desmin filaments is the primary pathogenic key event in EBS-MD striated muscle tissue. In keeping with this notion, the only reported histopathological study of cardiac muscle from an EBS-MD patient with dilated cardiomyopathy revealed a disruption of the regular desmin and plectin staining pattern at the level of Z-discs and intercalated discs as well as the presence of desmin-positive protein aggregates [2]. Furthermore, our previously reported desmin immogold EM analysis of patient 2 demonstrated the presence of subsarcolemmal and sarcoplasmic protein aggregates, which are composed of preformed but highly disordered desmin filaments [17]. Our new immunoblot studies further highlighted increased desmin protein levels in all EBS-MD samples indicating alterations in the overall desmin protein homeostasis. Thus, increased desmin protein levels in conjunction with the defective anchorage and spacing of preformed desmin filaments are the basis for the formation of subsarcolemmal and sarcoplasmic protein aggregates, which are composed of desmin, synemin-, and syncoilin IFs.

Previous studies in plectin and desmin knock-out mice convincingly demonstrated that the formation of endogenous plectin-desmin IF networks is pivotal for the normal organization, content, function and regulation of mitochondrial networks [7, 12, 22]. Hence, the observed changes in the subcellular distribution and amount of mitochondria as well as respiratory chain dysfunction in EBS-MD muscle are likely to be a direct consequence of the primary and omnipresent disruption of the plectin-desmin IF networks, rather than being an nonspecific secondary effect of muscle degeneration. Thus, our study strongly implies that the mitochondrial pathology is a fundamental pathogenic mechanism that contributes to the progressive and severe muscle damage in EBS-MD.

## Conclusion

Our study demonstrates that EBS-MD causing *PLEC* mutations, though leading to marked differences in the individual plectin protein expression pattern, all result in a desmin protein aggregate myopathy phenotype. Since plectin is the key cytolinker protein that regulates the structural and functional organization of the desmin filaments, the defective anchorage and spacing of assembled desmin filaments is the key pathogenetic event that triggers the formation of desmin protein aggregates as well as the secondary mitochondrial pathology.

### Ethics approval and consent to participate

This study was approved by the local Ethics committees of each participating institution. Written informed consent was obtained from all participants.

### Consent for publication

Consent for publishing personal data was obtained from patient 1.

### Availability of data and materials

The datasets supporting the conclusions of this article are included within the article and its additional file.

## Additional file

Additional file 1: Figure S1. Sanger confirmation of the identified *PLEC* mutations in patient 1. Plectin mutation analyses using genomic DNA derived from patient 1 and his parents compared to a healthy control individual presenting the maternally inherited non-frameshift deletion c.2264_2266delTCT and the paternally inherited frameshift deletion c.3119_3120delAA. (TIF 1670 kb)

## Abbreviations

AS: antiserum
COX: cytochrome oxidase
EBS-MD: epidermolysis bullosa simplex with muscular dystrophy
EBS-MD-MyS: epidermolysis bullosa simplex with muscular dystrophy with myasthenic features
EBS-PA: epidermolysis bullosa simplex with pyloric atresia
IF: intermediate filament
LGMD: limb girdle muscular dystrophy
mAb: mouse monoclonal antibody
SDH: succinate dehydrogenase
