# Supplementary figures and images for: Downstream effects of plectin mutations in epidermolysis bullosa simplex with muscular dystrophy
Source: Acta Neuropathol Commun. 2016 Apr 27;4:44. doi: 10.1186/s40478-016-0314-7 (PMC4847350; doi:10.1186/s40478-016-0314-7)

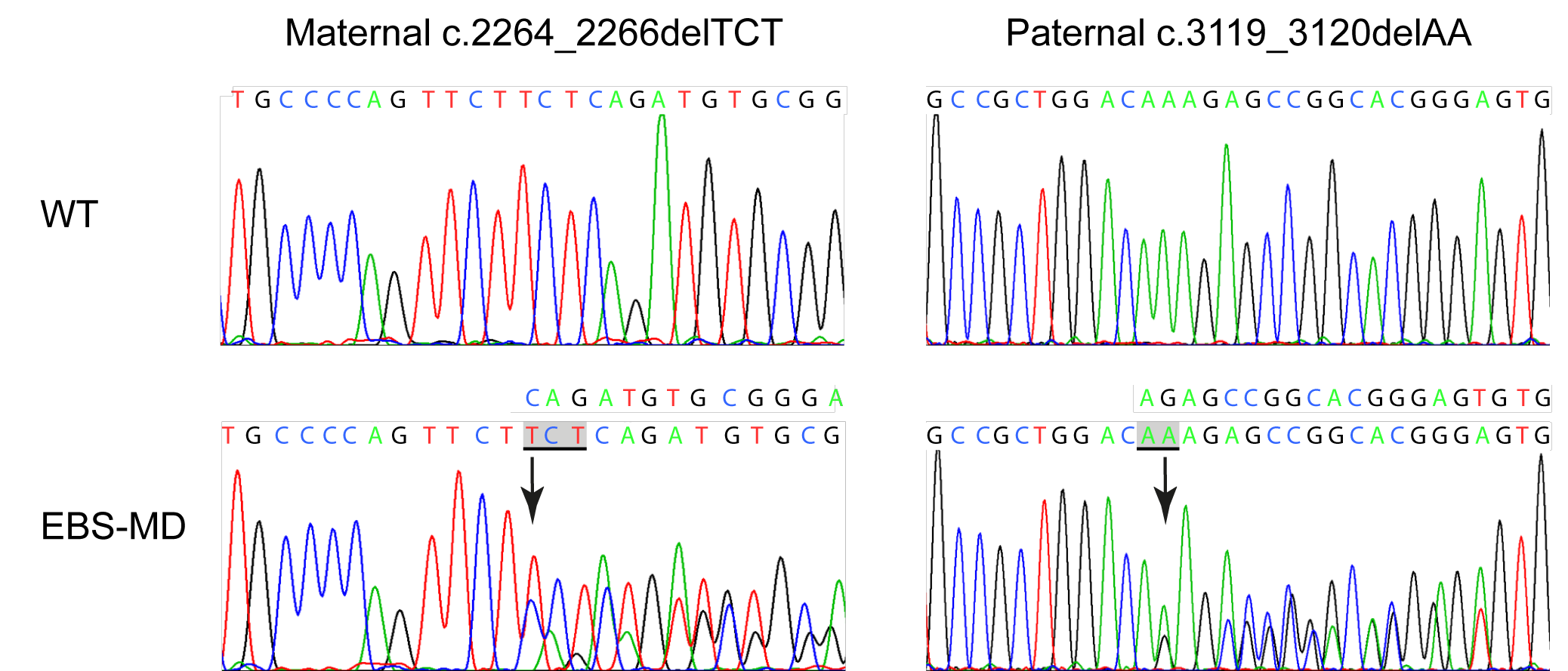

Supplement: Additional file 1: Figure S1. — Sanger confirmation of the identified PLEC mutations in patient 1. Plectin mutation analyses using genomic DNA derived from patient 1 and his parents compared to a healthy control individual presenting the maternally inherited non-frameshift deletion c.2264_2266delTCT and the paternally inherited frameshift deletion c.3119_3120delAA. (TIF 1670 kb) [file 40478_2016_314_MOESM1_ESM.tif]
